# Therapeutic potential of voltage-dependent potassium channel subtype 1.3 blockade in alleviating macrophage-related renal inflammation and fibrogenesis

**DOI:** 10.1038/s41420-025-02508-7

**Published:** 2025-05-05

**Authors:** Sha-sha Li, Yan Liang, Jia-wei Kong, Qi Zhang, Jing-rong Qian, Li-xia Yu, Qi-feng Liu

**Affiliations:** 1 Clinical Research & Lab Centre, Affiliated Kunshan Hospital of Jiangsu University, Kunshan, Jiangsu China; 2 Gusu School, Nanjing Medical University, The First People’s Hospital of Kunshan, Kunshan, Jiangsu China; 3 Department of Nephrology, Affiliated Kunshan Hospital of Jiangsu University, Kunshan, Jiangsu China

**Keywords:** Renal fibrosis, Chronic inflammation

## Abstract

Macrophage polarization and infiltration are notable characteristics of kidney injury and fibrosis. Although voltage-dependent potassium channel subtype (Kv) 1.3 is involved in macrophage-induced inflammation, its precise mechanism has not been elucidated. Therefore, this study aimed to explore the role of Kv1.3 in renal injury and macrophage polarization. Herein, mouse models of kidney injury were established through unilateral ureteral obstruction (UUO) and ischemia-reperfusion injury (IRI). For intervention, a selective Kv1.3 blocker, margatoxin (MgTx), was administered intraperitoneally. Blood and kidney samples were collected on days 3 and 7 following UUO surgery to evaluate renal Kv1.3 expression, kidney injury, macrophage polarization changes, cytokine levels, phosphorylation of extracellular signal-regulated kinase (ERK) and nuclear factor kappa-light-chain-enhancer of activated B (NF-κB). Kidney samples were also collected 24 h after IRI to assess kidney injury and evaluate renal Kv1.3 expression, as well as the phosphorylation levels of ERK and NF-κB. Histological analysis of MgTx-treated UUO and IRI mice revealed that Kv1.3 inhibition markedly alleviated renal damage induced by UUO and IRI, substantially reducing the levels of myofibroblast markers, specifically α-smooth muscle actin and transforming growth factor-β1. In UUO mice, Kv1.3 expression and proportions of monocyte-derived cells in peripheral blood and M1 macrophages notably increased but reversed after MgTx treatment, indicating diminished macrophage infiltration. Additionally, MgTx treatment downregulated various M1-related proinflammatory markers, including tumor necrosis factor-α, inducible nitric oxide synthase, and interleukin (IL)-1β, and upregulated M2-associated markers such as IL-10, arginase-1, and CD206. Moreover, Kv1.3 overexpression in THP-1 cells upregulated M1 macrophage markers and proinflammatory cytokines, enhanced their migratory ability. This indicates an increased polarization towards the M1 phenotype, which correlates with impaired renal tubular epithelial cells. Notably, Kv1.3 upregulation both in vivo and in vitro led to increased phosphorylation of ERK and NF-κB, possibly promoting M1 macrophage polarization. This study establishes Kv1.3 as a pivotal regulator of renal fibrosis and macrophage polarization, showing that its inhibition leads to reduced infiltration and migration of M1 macrophages, mitigation of renal injury via suppression of ERK/NF-κB signaling. Altogether, these findings suggest the potential of Kv1.3 as a promising therapeutic target for renal fibrosis.

## Introduction

Kidney injury and fibrosis are interrelated pathological processes resulting from inflammatory responses and maladaptive repair mechanisms, ultimately leading to progressive renal dysfunction. [1, 2]. Insults such as ischemia, infection, or nephrotoxicity can upregulate chemokines (e.g., CXCL1, CXCL8) and adhesion molecules (e.g., intercellular adhesion molecule-1, vascular cell adhesion molecule-1), which in turn recruit immune cells such as neutrophils and macrophages, promoting the release of pro-inflammatory cytokines (e.g., tumor necrosis factor [TNF]-α, interleukin [IL]-6). This cascade further damages endothelial cells, increases vascular permeability, and exacerbates renal tubular injury [3, 4]. Persistent inflammation leads to chronic damage to the renal parenchyma and a continuous cycle of injury and repair, leading to excessive deposition of the fibrous tissue, kidney function deterioration, and the progression of chronic kidney disease (CKD) [2]. The intricate relationship between inflammation and fibrosis is crucial for understanding the pathophysiology of kidney diseases and thereby developing effective therapeutic strategies aimed at mitigating renal disorders.

Macrophages are key mediators of the immune response, playing crucial roles in both the initiation and resolution of renal inflammation [5, 6]. They exhibit different polarization states, primarily categorized as M1 (proinflammatory) and M2 (anti-inflammatory) phenotypes, whose imbalance may lead to excessive inflammatory responses and tissue damage, exacerbating the severity of renal inflammation [6, 7]. However, despite the known involvement of macrophages in renal fibrosis, the underlying mechanisms associated with macrophage differentiation and polarization remain unelucidated.

Voltage-dependent potassium channel subtype (Kv) 1.3 is one of the major potassium channels in macrophages. Due to its potential function in modulating macrophage activity, Kv1.3 has attracted substantial attention. Reportedly, Kv1.3 can regulate the polarization state of macrophages, affecting the secretion of inflammation-associated cytokines, and thus, impacting inflammation and immune responses [8, 9]. Moreover, it can modulate intracellular calcium levels in macrophages, thereby influencing cell activation status and function. It has also been shown to affect the macrophage migratory by regulating cell membrane potential and intracellular calcium influx. Margatoxin (MgTx), a short peptide extracted from scorpion venom, is a high-affinity and selective inhibitor of the Kv1.3 channel [10]. Recent studies have shown that MgTx alleviates lipopolysaccharide (LPS) or LPS+ D-galactosamine-induced liver inflammation and acute liver injury by inhibiting Kv1.3-dependent macrophage migration [11]. Furthermore, MgTx mitigates alcoholic liver injury by reducing the infiltration of LY6C^hi^ macrophages into the liver [10]. Beyond its anti-inflammatory properties, MgTx also demonstrates significant antifibrotic effects. By suppressing the polarization of macrophages towards the pro-inflammatory M1 phenotype, MgTx reduces the production of pro-inflammatory cytokines, alleviating CCl4-induced liver fibrosis [12]. In addition, MgTx has been shown to significantly inhibit leukocyte counts and the progression of renal fibrosis in a rat model of chronic renal failure following 5/6 nephrectomy [13]. These findings highlight the therapeutic potential of MgTx in regulating macrophage activity and combating fibrotic diseases. Activation of extracellular signal-regulated kinase (ERK), a mitogen-activated protein kinase (MAPK), promotes M1 polarization of macrophages [14], leading to the downregulation of anti-inflammatory cytokines such as IL-10, and thereby affecting their roles in inflammatory responses and tissue repair [6]. Similarly, activation of nuclear factor kappa-light-chain-enhancer of activated B (NF-κB) promotes M1 polarization of macrophages, upregulating proinflammatory cytokines (such as TNF-α and IL-1β) [15]. Hence, the ERK and NF-κB signaling in macrophage polarization exhibit mutual interactions and reciprocal regulation, affecting the polarization status and inflammatory responses by modulating cytokine expression and changes in cellular function.

However, the role of Kv1.3 in macrophage polarization in kidney diseases and the potential mechanisms have not been reported. Therefore, this study aimed to elucidate the role of Kv1.3 blockade against renal inflammation and injury both in vitro and in vivo, along with the underlying mechanism involved. The findings of this study may provide insights into innovative therapeutic strategies targeting macrophage polarization to treat renal inflammation and fibrosis and improve outcomes for patients with CKD.

## Results

### Kv1.3 inhibition alleviated renal fibrogenesis and improved morphological impairments caused by unilateral urethral obstruction (UUO) and ischemia/reperfusion injury (IRI) in mice

**Fig. 1:**
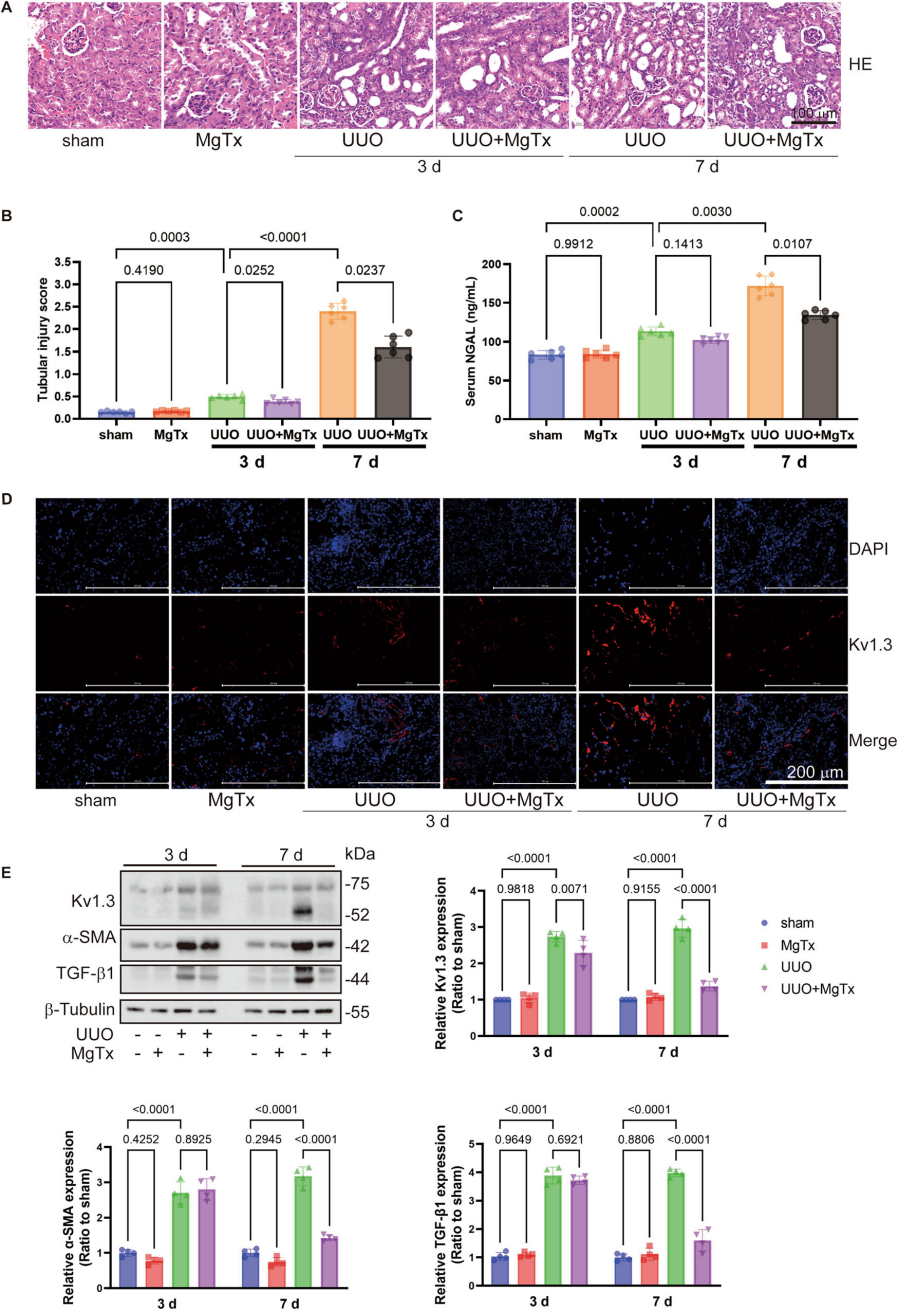
Kv1.3 inhibition alleviates renal damage in UUO mice. **A** Representative images of HE staining. **B** The tubular injury score was evaluated based on pathological observations from HE staining. Data were expressed as mean ± SD (n = 6). **C** Serum NGAL levels in mice were determined to assess injury to renal tubular epithelial cells. Data were expressed as mean ± SD (n = 6). **D** Representative images of Kv1.3 (red) expression in UUO mice as detected by immunofluorescence staining. **E** The immunoblot of Kv 1.3, TGF-β1, α-SMA and β-Tubulin. Densitometry analysis of Kv 1.3, TGF-β1 and α-SMA protein levels, and normalized to β-Tubulin. Data were expressed as mean ± SD (n = 4).

**Fig. 2:**
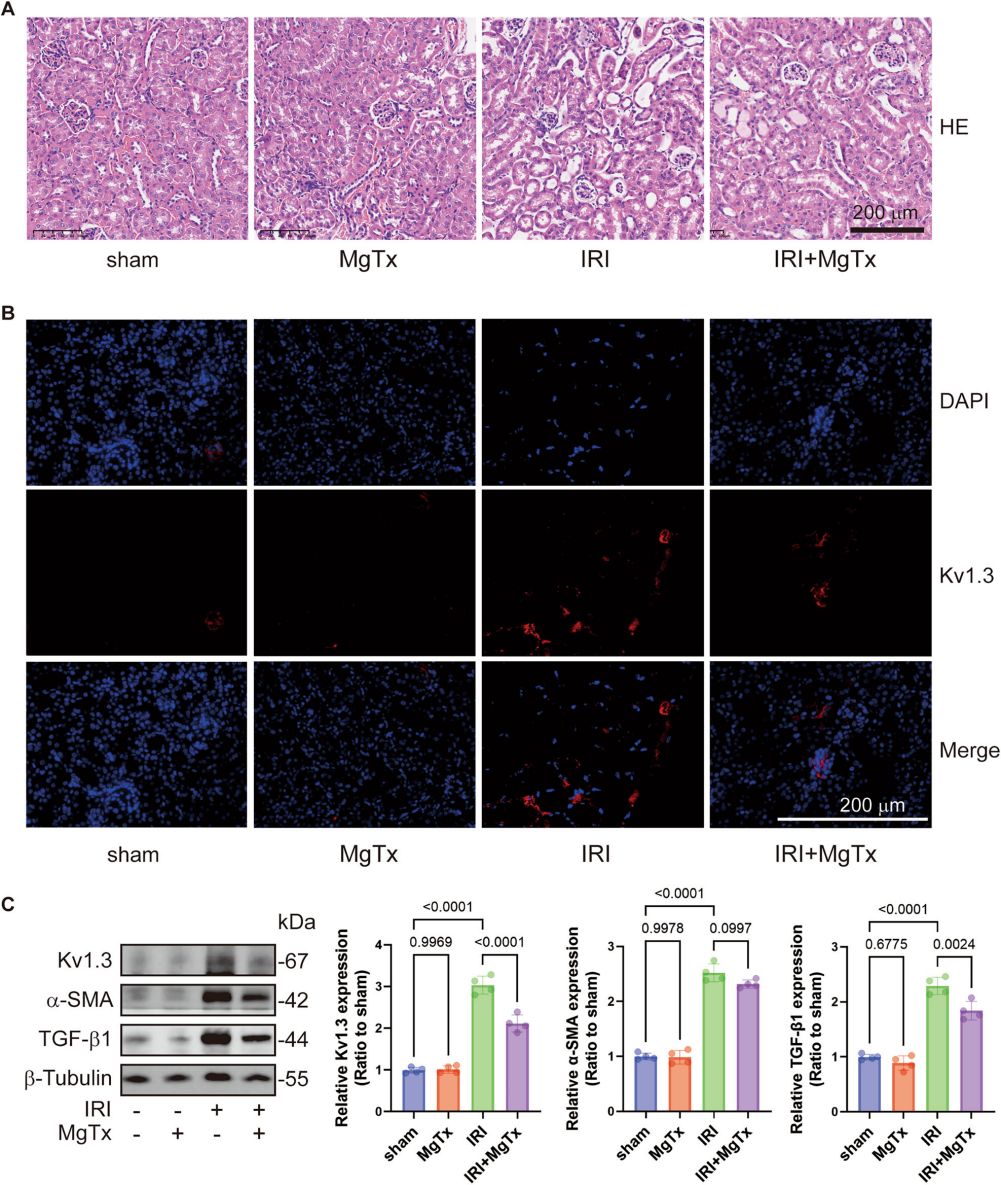
Kv1.3 inhibition alleviates renal damage in IRI mice. **A** Representative images of HE staining. **B** Representative images of Kv1.3 (red) expression in IRI mice as detected by immunofluorescence staining. **C** The immunoblot of Kv 1.3, TGF-β1, α-SMA and β-Tubulin. Densitometry analysis of Kv 1.3, TGF-β1 and α-SMA protein levels, and normalized to β-Tubulin. Data were expressed as mean ± SD (n = 4).

Hematoxylin and eosin (HE) staining results (Fig. 1A) revealed notable renal tissue damage in UUO mice on postoperative days 3 and 7. This damage included the loss of brush borders of tubular cells, disruption, congestion, and dilation of tubules, interstitial edema, and inflammatory cell infiltration. Furthermore, the structural damage to the renal tissue progressively worsened with increasing obstruction duration. Notably, UUO-induced tubular cell damages were alleviated after MgTx administration, especially on postoperative day 7 (Fig. 1A). Renal injury scores based on HE staining (Fig. 1B) showed more severe injury on day 7 than day 3, but MgTx treatment significantly reduced injury scores on both day 3 and 7. Additionally, serum neutrophil gelatinase-associated lipocalin (NGAL), an early biomarker of renal injury, were elevated in UUO mice, particularly on day 7, but were significantly lowered by MgTx treatment (Fig. 1C), further corroborating the HE staining results. Moreover, the HE results from the IRI model also confirmed the role of MgTx in reducing renal injury (Fig. 2A), highlighting its potential therapeutic benefits.

To investigate the role of Kv1.3 inhibition on renal interstitial fibrosis, different markers associated with renal fibrosis were examined in mice subjected to UUO and IRI, both with or without MgTx administration. Western blotting analysis (Figs. 1E and 2C) demonstrated significant elevations in renal tubular injury marker α-smooth muscle actin (SMA) and transforming growth factor (TGF)-β1 in both UUO and IRI models. Following MgTx treatment, the levels of SMA and TGF-β1 in the kidneys of UUO mice were markedly reduced, particularly on day 7. Similarly, MgTx administration in IRI mice resulted in significant decreases in the levels of α-SMA and TGF-β1. Collectively, these findings suggest that both UUO and IRI induce renal fibrosis, which can be effectively reversed by MgTx treatment. This indicates that MgTx provides protective effects against renal interstitial damage caused by UUO and IRI.

### Kv1.3 inhibition mitigated the UUO- and IRI-induced increase in renal Kv1.3 expression in mice

Immunofluorescence (IF) staining (Figs. 1D and 2B) revealed that the interstitial Kv1.3-positive area in the kidney was significantly higher in UUO and IRI mice compared with that in the sham group. Moreover, MgTx treatment led to a notable reduction in the Kv1.3-positive area in both UUO and IRI mice. Consistent with these findings, western blotting analysis (Fig. 1E) demonstrated that UUO induced elevated Kv1.3 levels on both day 3 and 7 compared to the sham group. MgTx treatment significantly downregulated Kv1.3 expression in the renal tissue of UUO mice on both days. Similarly, IRI mice exhibited increased Kv1.3 levels than the sham group, and MgTx effectively decreased Kv1.3 expression in the renal tissue of IRI mice as well (Fig. 2C). These results indicate that MgTx can effectively inhibit Kv1.3 expression in the renal tissue of both UUO and IRI mice, highlighting its potential value in the treatment of kidney injury.

### Kv1.3 inhibition reduced monocyte proportion in peripheral blood in UUO mice

**Fig. 3:**
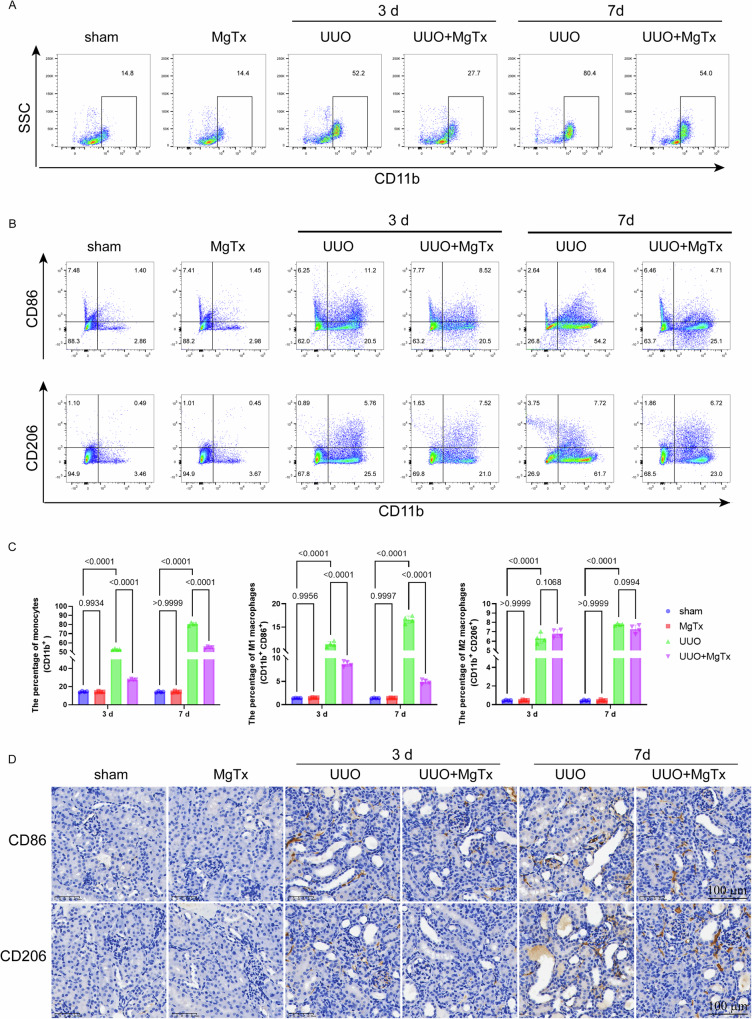
Targeting Kv1.3 channel inhibits polarizing M1 macrophages in UUO mice. **A** Respective FCM analysis the percentage of monocytes in peripheral blood of experimental mice. **B** Respective FCM analysis of M1 (CD11b^+^CD86^+^) and M2 (CD11b^+^CD206^+^) macrophages changes in experimental mice. **C** Analysis of the proportion of monocytes in peripheral blood and the proportion of M1 (CD11b^+^CD86^+^) and M2 (CD11b^+^CD206^+^) macrophages changes in renal tissue. **D** Representative images of CD86 and CD206 as detected by immunohistochemical staining.

The proportion of mononuclear cells (MNCs) in UUO mice was markedly increased up to 52.2% at postoperative day 3 compared with that in the sham group, with a continued rise observed with increasing obstruction period. By day 7, the proportion of MNCs in UUO mice rose to 80.4% (Fig. 3A, C).

Following MgTx administration, the proportion of MNCs decreased to 27.7% and 54% at days 3 and 7, respectively, indicating the amelioration of the inflammatory response in UUO mice by MgTx. Furthermore, MgTx administration did not substantially affect the proportion of MNCs in sham-operated mice, suggesting no notable toxic effects were exerted (Fig. 3A, C).

### Targeting Kv1.3 inhibited M1 polarization of macrophages and downregulated M1-related markers in mice

The flow cytometric analysis of macrophage subtypes within the renal tissue of UUO mice models (Fig. 3B, C) revealed a notable increase in the proportions of both M1 (CD11b^+^CD86^+^) and M2 (CD11b^+^CD206^+^) macrophages in the kidney tissue of UUO mice, compared with that in the sham group, which positively correlated with the obstruction period (day 3–7). These findings underscore the pivotal role of macrophages in the pathophysiological processes involved in UUO-induced kidney inflammation and injury. MgTx treatment decreased the proportion of M1 macrophages in the kidney tissue of UUO mice on postoperative day 3, with a significant decrease observed by day 7. Additionally, no notable change in the proportion of M2 macrophages was observed, whether on postoperative day 3 or 7. Notably, MgTx did not alter the macrophage population dynamics in the normal renal tissue. The results of the immunohistochemistry analysis (Fig. 3D) validate the above findings.

**Fig. 4:**
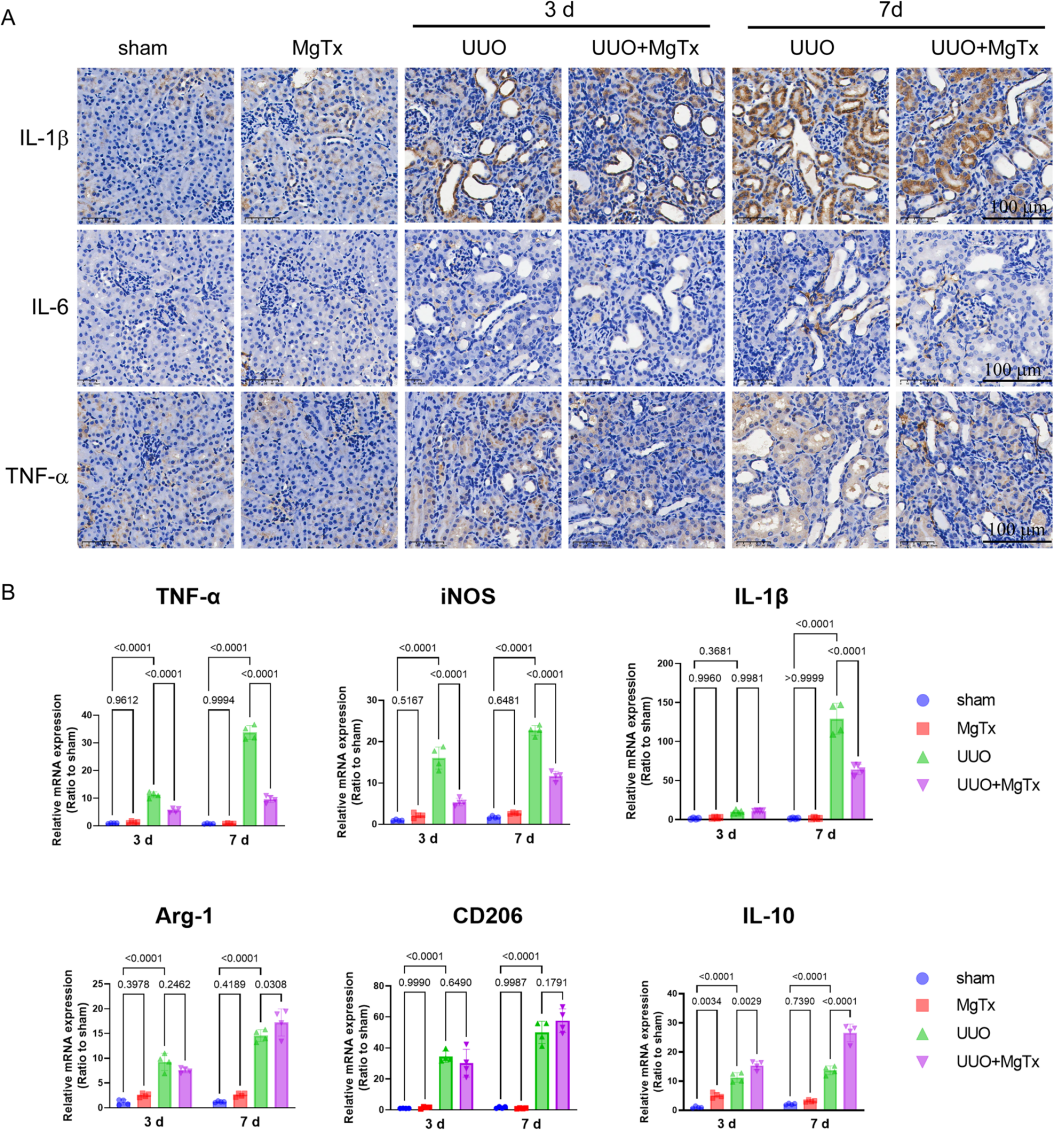
Targeting Kv1.3 channel reduces pro-inflammatory cytokines in UUO mice. **A** Representative images of IL-1β, IL-6 and TNF-α as detected by immunohistochemical staining. **B** Quantitative analysis of mRNA expression levels of M1 and M2-related cytokines in UUO mice. Data were expressed as mean ± SD (n = 4).

To further confirm the role of MgTx in the alteration of macrophage polarization, the expression of M1- and M2-associated genes were examined through quantitative real-time PCR (RT-qPCR) (Fig. 4B). UUO markedly increased the expression of M1-related proinflammatory markers (such as TNF-α, iNOS, and IL-1β), whereas M2-related markers (such as arginase-1 [Arg-1], CD206, IL-10) showed a moderate increase in their expression. MgTx treatment significantly downregulated the expression of TNF-α and iNOS on days 3 and 7 and decreased IL-1β expression on day 7. In contrast, the expression of M2-related markers (Arg-1, CD206, and IL-10) was significantly increased on days 3 and 7 after UUO surgery. However, after treatment with MgTx, the expression of IL-10, Arg-1, and CD206 was increased on day 7, although the increase in CD206 was not statistically significant. Additionally, immunohistochemistry results showed that UUO increased the expression of M1-related markers such as IL-1β, IL-6, and TNF-α, whereas MgTx treatment reduced their expression, particularly on postoperative day 7 (Fig. 4A). Collectively, these findings show that Kv1.3 inhibition effectively mitigates M1-like polarization of macrophages in vivo.

### Kv1.3 overexpression enhances M1 polarization and migration, exacerbating damage in renal tubular epithelial cells

**Fig. 5:**
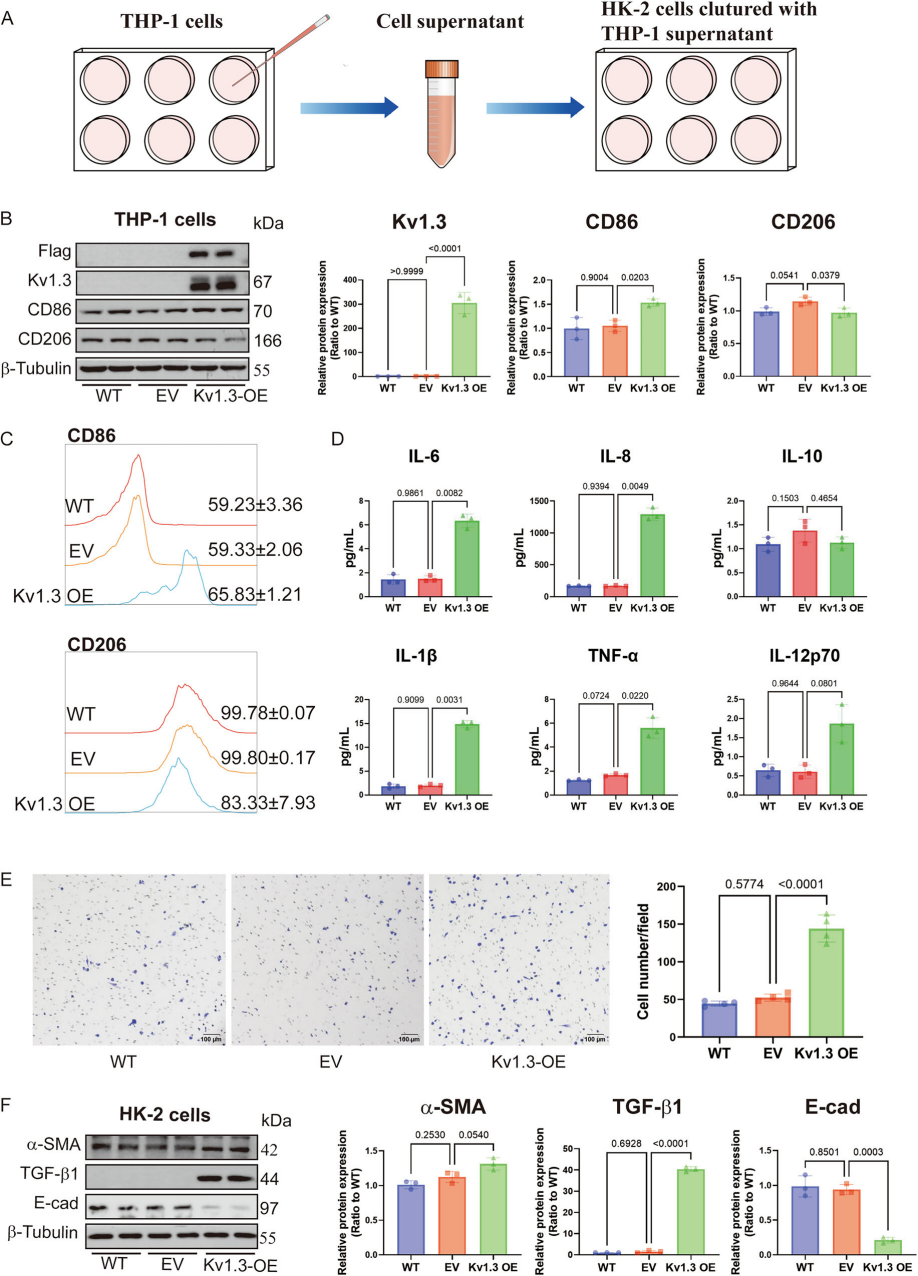
Kv1.3-overexpression induced M1 macrophages, Aggravated damage of renal tubular epithelial cells. **A** Experimental process flow chart. **B** Western blotting analysis of Kv1.3, CD86, and CD206 levels after transfection with LV-Kv1.3 in THP-1 cells. **C** Respective FCM analysis of CD86 and CD206 expression in Kv1.3-overexpressing THP-1 cells. **D** CBA analysis of the cytokines (IL-6, IL-8, IL-10, IL-1β, TNF-α, IL-12p70) alterations in supernatant of THP-1 cells. **E** Representative migration images and statistical analysis of Transwell assay. Data were expressed as mean ± SD (n = 4). **F** Respective western blotting image and densitometry analysis of α-SMA, TGF-β1 and E-cad levels, and normalized to β-Tubulin. Data were expressed as mean ± SD (n = 3).

To identify the relationship between Kv1.3, macrophage polarization, migration and damage to renal tubular epithelial cells, Kv1.3 was overexpressed in THP-1 cells, which were then co-cultured with human renal proximal tubular cells (HK-2). Results showed that Kv1.3-overexpressing THP-1 cells exhibited a notable increase in the expression of human M1 macrophage marker CD86, while the expression of M2 macrophage marker CD206 was suppressed (Fig. 5B), which was further confirmed by flow cytometry (Fig. 5C). Moreover, Kv1.3 overexpression induced marked increase in M1-related cytokine levels (such as IL-6, IL-8, IL-1β, TNF-α, and IL-12p70) in the supernatant of THP-1 cells (Fig. 5D). Additionally, Kv1.3 overexpression enhanced the migratory capacity of THP-1 cells (Fig. 5E). Notably, after 48 h of incubation with the supernatant from THP-1 cell, the levels of TGF-β1 and α-SMA in HK-2 cells significantly increased, whereas E-cadherin (E-cad) levels markedly decreased (Fig. 5F). These findings collectively indicate that Kv1.3 overexpression promotes M1 polarization, enhances the migratory capacity, and exacerbates damage to renal tubular epithelial cells.

### The ERK/NF-κB pathway is involved in Kv1.3 overexpression-induced M1 macrophage polarization both in vitro and in vivo

**Fig. 6:**
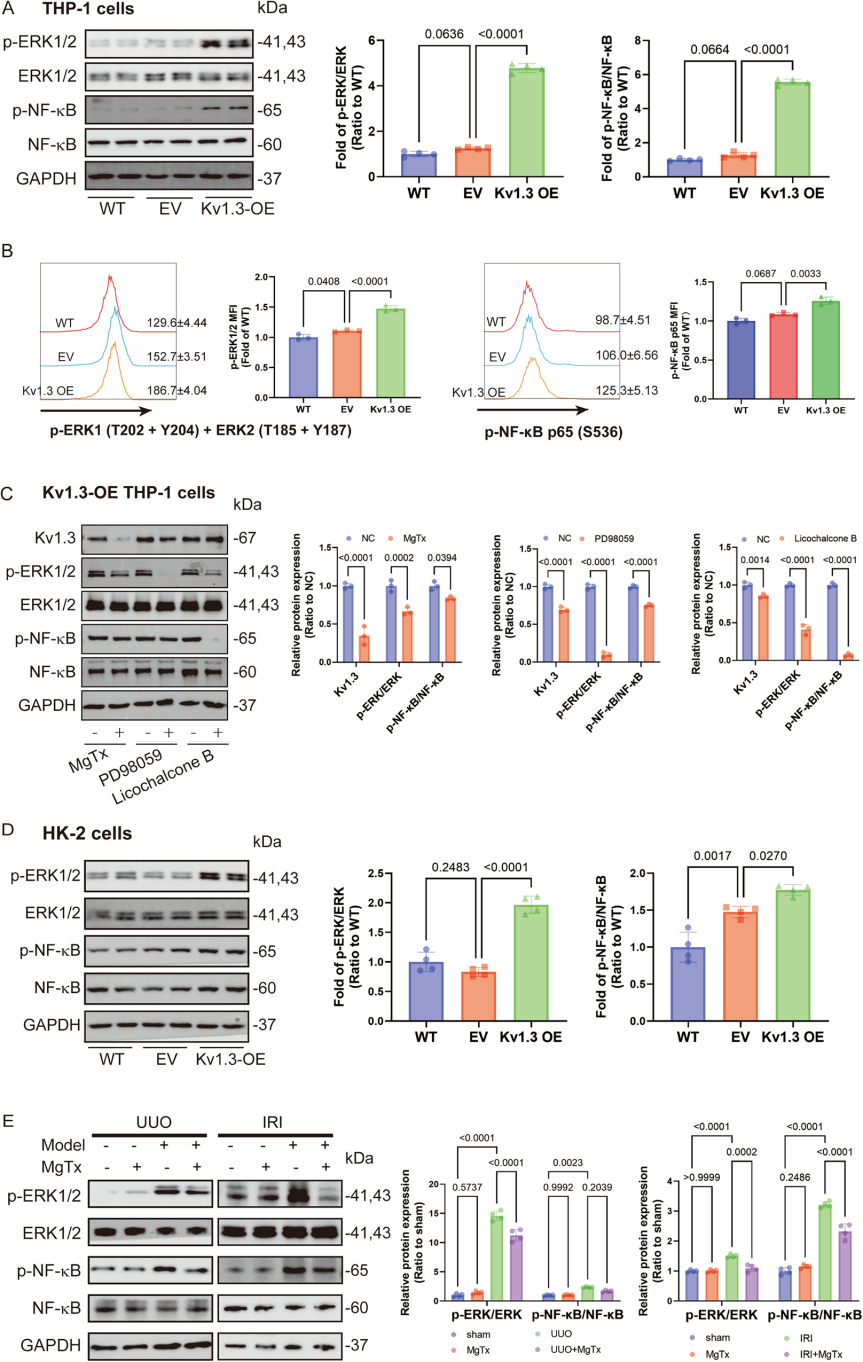
ERK/NF-κB signaling is involved in Kv1.3-related M1 macrophages. **A** Respective western blotting image and densitometry analysis of ERK/NF-κB signaling after transfection with LV-Kv1.3 in THP-1 cells. Data were expressed as mean ± SD (n = 4). **B** The phosphorylation signal data are depicted in the form of stacked histogram overlaid plots. The mean fluorescence intensity (MFI) of the ERK1/2 and NF-κB p65 phosphorylation signal was expressed as the mean ± SD (n = 3). **C** Effects of MgTx, PD98059, and Licochalcone B on Kv1.3 and ERK/NF-κB signaling in LV-Kv1.3-transduced THP-1 cells were analyzed by western blotting. Data were expressed as mean ± SD (n = 3). **D** Respective western blotting image and densitometry analysis of ERK/NF-κB signaling in HK-2 cells co-cultured with the supernatant from LV-Kv1.3-transduced THP-1 cells for 48 h. Data were expressed as mean ± SD (n = 4). **E** Respective western blotting image and densitometry analysis of ERK/NF-κB signaling in mice subjected to UUO (7 days post-surgery) and IRI. Data were expressed as mean ± SD (n = 4).

The changes in phosphorylation of the ERK/NF-κB pathway and its association with M1 polarization were explored in THP-1 cells. Results showed that Kv1.3 overexpression significantly enhanced the phosphorylation of ERK1 (T202 + Y204) and ERK2 (T185 + Y187) and NF-κB (S536) in THP-1 cells (Fig. 6A, B). Additionally, after 48 h of incubation with the supernatant from THP-1 cells, there is a notable increase in ERK1/2 and NF-κB phosphorylation in HK-2 cells (Fig. 6D). In Kv1.3-overexpressing THP-1 cells, treatment with the Kv1.3 inhibitor MgTx resulted in decreased Kv1.3 levels, which was accompanied by reduced ERK1/2 and NF-κB phosphorylation (Fig. 6C). Conversely, when ERK1/2 phosphorylation was inhibited with PD98059, Kv1.3 expression was decreased along with a reduction in NF-κB phosphorylation (Fig. 6C). Furthermore, treatment with Licochalcone B, known to inhibit NF-κB phosphorylation, led to a decrease Kv1.3 levels and a reduction in ERK1/2 phosphorylation (Fig. 6C). Collectively, these results highlight a complex regulatory interplay among Kv1.3, ERK1/2, and NF-κB within THP-1 cells.

To further verify the role of the ERK/NF-κB pathway in renal fibrogenesis, in vivo phosphorylation levels of ERK1/2 and NF-κB were assessed by western blotting. Consistent with the in vitro findings, phosphorylation of ERK1/2 and NF-κB were markedly increased in both UUO mice on postoperative day 7 and IRI mice, which was partially ameliorated by MgTx treatment (Fig. 6E). Altogether, these results show that Kv1.3 upregulation can promote ERK/NF-κB phosphorylation, thereby facilitating to M1 macrophage polarization in both in vivo models of UUO and IRI, as well as in vitro studies using Kv1.3-overexpressing cells.

## Discussion

This study showed that pharmacological inhibition of Kv1.3 with MgTx significantly alleviated renal injury induced by UUO and IRI by reducing the migration and infiltration of M1 macrophage. This reduction in macrophage subsequently diminished renal fibrosis, as indicated by lower tubular injury scores, decreased serum NGAL concentrations, and reduced levels of fibrotic markers such as α-SMA and TGF-β1, along with increased expression of E-cad. The findings provide strong evidence that Kv1.3 blockade can modulate macrophage polarization, thereby mitigating renal inflammation and fibrosis, making Kv1.3 in macrophage an attractive therapeutic target for renal inflammation and injury.

Kv1.3 has been reported to be involved in the regulation of macrophage polarization [16] and immune responses [17]. Reportedly, angiotensin (Ang)II increased the expression of Kv1.3 in the aorta and peritoneal macrophages of mice. Inhibition Kv1.3 effectively corrected AngII-induced macrophage infiltration and endothelial dysfunction in the small mesenteric arteries and/or aorta [18]. Additionally, MgTx treatment has been shown to alleviate damaged liver tissues by reducing δ-catenin-related macrophage infiltration in a mice model of acute liver injury [11]. Moreover, a study showed that MgTx alleviated CCl4‑induced hepatic fibrosis in mice by reducing M1 macrophage polarization and proinflammatory cytokine secretion [12]. In a mouse model of CKD, increased Kv1.3 expression was noted, and both pharmacological inhibition and genetic ablation affirmed its critical role in uremia-induced vascular smooth muscle cell calcification [19]. Furthermore, a rise in macrophages proportions contributed to the progression of tubulointerstitial renal fibrosis in UUO rat kidneys, with MgTx treatment reducing interstitial inflammation and renal fibrosis, strongly suggesting the potential of Kv1.3-targeted therapeutic approach for treating renal fibrosis [20]. Altogether, these studies indicate that Kv1.3 may enhance macrophage polarization toward the M1 phenotype, thereby facilitating inflammatory responses and organ fibrosis. However, these findings remain unvalidated in kidney diseases. Increased Kv1.3 levels correlate with elevated macrophage numbers in the kidneys, which, in turn, exacerbate the progression of CKD; however, further mechanistic investigations are lacking. Therefore, in this study, the correlation between increased Kv1.3 expression and M1 macrophage polarization was investigated in the kidneys of UUO mice. The results showed that elevated Kv1.3 expression in the kidneys of UUO mice was positively associated with the increased proportion of M1 macrophages and enhanced production of proinflammatory cytokine. Additionally, flow cytometry further indicated that Kv1.3 overexpression in THP-1 cells led to M1 polarization, which was characterized by increased CD86 and decreased CD206 expression, along with enhanced imgration capacity and elevated levels of proinflammatory cytokines such as IL-1β, IL-6, and iNOS in the cell supernatant. Furthermore, co-culturing HK-2 cells with supernatants from Kv1.3-overexpressing THP-1 cells exacerbated the inflammatory response and cellular damage in renal tubular epithelial cells. Overall, the findings confirmed the crucial role of Kv1.3 in promoting M1 macrophage polarization and subsequent migration and inflammatory responses. Previous studies on Kv1.3 have mainly focused on pulmonary, hepatic or cardiovascular fibrosis [21]; in contrast, this study elucidates the role of Kv1.3 in renal fibrosis, highlighting its potential as a therapeutic target in kidney diseases.

Reportedly, Kv1.3 blockade inhibited proinflammatory microglial activation and cytokine production by targeting the NF-κB signaling [22]. In one study, Kv1.3 overexpression increased IL-6 and TNF-α expression and phosphorylation of ERK1/2 and NF-κB in macrophages. This suggests that Kv1.3 is a crucial membrane protein involved in oxidized low-density lipoprotein-induced inflammation in RAW264.7 macrophages during atherosclerosis via the ERK/NF-κB pathway [23]. Notably, 5-(4-phenoxybutoxy) (PAP-1), a selective pharmacological Kv1.3 blocker, reduces NF-κB p65 and p50 activation, thus, facilitating the polarization of microglia into an M2-like phenotype [24]. In a mouse model of asthma, Kv1.3 expression and Kv current intensity were markedly increased. PAP-1 treatment suppressed the activation of the ERK/NF-κB pathway, thereby alleviating asthma in the neutrophilic asthma model [25]. These findings suggest that ERK and NF-κB act as downstream regulators of Kv1.3.

Numerous studies have reported the involvement of ERK and NF-κB in the pathophysiology of kidney diseases. Specifically, activation of the MAPK/ERK pathway has been shown to enhance autophagy in renal tubular cells, while the inhibition of ERK activation has been reported to reduce renal fibrosis and improve kidney function. Fibroblast activation has been shown to be accompanied by increased the phosphorylation of ERK in primary cultured fibroblasts [26] and renal tubular cells [27]. Targeting NF-κB signaling within renal tubular cells has also been found to mitigate kidney inflammation associated with proximal tubular autophagy deficiency and the ablation of autophagy protein 5 gene [28]. Additionally, ERK phosphorylation in renal tissues and tubular cells has been associated with IRI-mediated deterioration of renal function and increased production of reactive oxygen species [29]. However, to date, no studies have established a correlation between the activation of the ERK/NF-κB pathway and Kv1.3-mediated polarization of M1 macrophages in renal diseases. This makes the current study the first to investigate the mechanisms underlying Kv1.3-mediated modulation of macrophage behavior. Notably, Kv1.3 overexpression in THP-1 cells led to the activation of the ERK/NF-κB signaling, which facilitates M1 polarization. Moreover, the supernatant from Kv1.3-overexpressing THP-1 cells was able to activate ERK/NF-κB signaling in HK-2 cells. In Kv1.3-overexpressing THP-1 cells, treatment with MgTx resulted in decreased Kv1.3 levels and a corresponding reduction in the phosphorylation of ERK1/2 and NF-κB. Furthermore, treatment with the ERK1/2 inhibitor PD98059 led to decreased phosphorylation of ERK1/2, which were associated with reductions in both Kv1.3 and NF-κB phosphorylation levels. Conversely, treatment with the NF-κB inhibitor Licochalcone B diminished NF-κB phosphorylation levels, which was similarly linked to decreases in Kv1.3 and ERK1/2 phosphorylation levels. These findings suggest a reciprocal regulatory relationship between Kv1.3 and the ERK1/2 and NF-κB signaling pathways in THP-1 cells. In vivo experiments further corroborated that UUO and IRI induced an increase in Kv1.3 expression, which was associated with the activation of ERK/NF-κB signaling. Additionally, MgTx treatment was found to inhibit both Kv1.3 expression and ERK/NF-κB activation.

Regardless of significant insights, this study has some limitations. The animal model used in this study, although informative, may not fully replicate the complexity of renal fibrosis in humans. Additionally, the study focused exclusively on the ERK/NF-κB pathway as a critical mediator; however, given the intricate interplay of various disease mechanisms, other pathways may also play important roles and warrant further investigation. Future research should focus on validating the therapeutic potential of Kv1.3 blockade in patients with renal fibrosis. Furthermore, exploring other signaling pathways that may be associated with Kv1.3 is important to uncover additional therapeutic targets. Investigating the role of Kv1.3 in other kidney injury models could also provide a broader understanding of its function. It is important to note that, despite MgTx’s high selectivity for Kv1.3 (Kd = 11.7 pM), challenges related to its immunogenicity and membrane permeability remain. Addressing these limitations may require chemical modifications or humanization of MgTx to minimize immune rejection. Furthermore, the development of nanodelivery systems or small molecule analogs could enhance its capacity to penetrate the blood-brain barrier [21, 30]. Finally, it is important to highlight that most existing data are based on animal models, underscoring the need for further clinical trials to validate the efficacy and safety of Kv1.3 inhibitors for use in human diseases.

In conclusion, this study highlights the critical role of Kv1.3 in renal fibrosis and macrophage polarization. Herein, inhibiting Kv1.3 effectively reduced M1 macrophage infiltration and renal fibrosis, primarily by suppressing the ERK/NF-κB signaling. Although further research is warranted, these findings suggest Kv1.3 as a promising therapeutic target for developing therapeutic strategies for renal fibrosis. By modulating macrophage polarization, Kv1.3 inhibitors could potentially reduce renal injury and improve kidney function in patients with CKD; hence, developing Kv1.3-targeted therapies can be a novel treatment option, potentially improving patient outcomes.

## Materials and methods

### Reagents and antibodies

MgTx, PD98059 and Licochalcone B were obtained from MedChemExpress (HY-P1280, HY-12028 and HY-N0373, respectively). The following antibodies were used for western blotting: anti-Kv1.3 from Proteintech (14079-1-AP) and Santa Scuz (sc-398855), anti-TGF-β1, anti-α-SMA, anti-phosphorylated ERK1 (p-ERK)1 (T202/Y204) + ERK2 (T185/Y187), anti-phosphorylated NF-κB (p-NF-κB) p65 (S529) from HuaBio (HA721143, ET1607-53, ET1610-13, ET1604-27), anti-ERK1/2 from Boster (BM4326), anti-NF-κB from CST (#8242), anti-GAPDH from Santa Cruz (sc-166574) and anti-β-Tubulin from Abcam (ab179513). Lentivirus (LV) overexpressing Kv1.3 (LV-Kv1.3) was designed and synthesized by GENECHEM CO. (Shanghai, China). The Mouse Organ Tissue Single Nucleated Cell Isolate Kit was obtained from Solarbio (Beijing, China). For the dissociation of the mouse kidney, the Multi Tissue Dissociation Kit and gentleMACS C Tubes were obtained from Miltenyi Biotec Inc. PerCP/Cyanine5.5 anti-mouse/human CD11b (101227), phycoerythrin anti-mouse CD86 (105007), APC anti-mouse CD206, FcRblock anti-mouse CD16/32 (101319) antibodies, along with fixation buffer (420801) and intracellular staining permeabilization wash buffer (421002) were obtained from BioLegend, Inc. Fast Pure Cell/Tissue Total RNA Isolation Kit, HiScript III RT SuperMix for qPCR (+gDNA wiper), and Taq Pro Universal SYBR qPCR Master Mix were obtained from Vazyme (Nanjing, China). Mouse Neutrophil Gelatinase-Associated Lipocalin (NGAL) ELISA Kit (E-EL-M0828) was obtained from Elabscience Biotechnology Co., Ltd. (Wuhan, China).

### UUO-induced renal injury mouse model

Forty-eight male C57BL/6 mice (4–6 weeks old, weight = 20 ± 2 g) were purchased from Hangzhou Ziyuan Experimental Animal Technology Co., Ltd., and randomly divided into the following four groups: sham-operated (sham), sham-operated+MgTx (MgTx), UUO, and UUO+MgTx. The UUO model was constructed by ligating the left ureter as previously described [31, 32]. In the sham group, the ureter was not dissected. MgTx was prepared with sterile phosphate-buffered saline (PBS) solution at a concentration of 300 nM, and it was injected intraperitoneally (100 μL/20 g qd) after surgery. Other groups were administered an equal amount of saline intraperitoneally. After anesthetizing, whole blood (EDTA-K_2_-anticoagulated) and tissue samples from each group were collected on days 3 and 7 post surgery, respectively.

### IRI-induced acute renal injury mouse model

Acute kidney injury was induced in forty-eight male C57BL/6 mice through IRI. The mice were randomly assigned to one of four groups: sham-operated (sham), sham-operated+MgTx (MgTx), IRI, and IRI+MgTx. After anesthetization, the mice underwent unilateral renal pedicle clamping for 60 min to induce ischemia while maintaining a constant body temperature of 37 °C. Following the clamping period, the renal pedicle clamps were released to initiate reperfusion. For MgTx treatment, MgTx was administered intraperitoneally before surgical procedure at the same dosage as previously. Twenty-four hours after surgery, the kidneys were harvested for further analysis.

### HE and IF staining

The kidney tissue was fixed in 4% paraformaldehyde, embedded in paraffin, and cut into 4-μm-thick sections. After dewaxing and dehydration, all sections were subjected to HE staining. For IF staining, sections underwent antigen retrieval in a boiled citrate-buffered solution (pH 6.0) for 30 min. Following this, sections were blocked with 10% bovine serum albumin and incubated with primary antibodies (Alexa Fluor 488^®^-labeled F4/80 and non-conjugated rabbit anti-Kv1.3) at 4 °C overnight. For Kv1.3, sections were counterstained with Alexa Fluor^®^ 647-conjugated anti-rabbit IgG antibody. Finally, the nuclei were stained with 4′,6-diamidino-2-phenylindole, and the sections were visualized by confocal microscopy (ZEISS LSM900, Germany). All processes were conducted under dark conditions. In the experiments, all negative controls were substituted with PBS in place of the primary antibody.

### Tubular injury assessment

Tubular injury was evaluated using HE staining, following the previously described method. Assessment involved grading tubular dilatation, epithelial simplification, and brush border loss across 15 randomly selected, non-overlapping fields at ×200 magnification. Lesions were scored on a scale from 0 to 4: 0 indicates normal; 1 represents mild injury (less than 25% of the cortex affected); 2 signifies moderate injury (25–50% of the cortex affected); 3 denotes severe injury (50–75% of the cortex affected); and 4 indicates extensive damage (more than 75% of the cortex affected) [33].

### Measurement of serum NGAL levels in mice

Serum NGAL levels in mice were determined using ELISA kit to evaluate renal tubular epithelial cell injury. The absorbance was measured at 450 nm with a microplate reader(Molecular Devices, USA).

### Cell culture, transfection, and treatments

Macrophages derived from the human monocytic leukemia THP-1 cell line were cultured in the Roswell Park Memorial Institute (RPMI) 1640 medium, supplemented with 10% fetal bovine serum (FBS). Renal proximal tubular epithelial HK-2 cells were cultured in the Dulbecco’s modified Eagle’s medium (DMEM), supplemented with 10% FBS.

To examine the association between Kv1.3-related macrophage polarization and renal tubular epithelial cells, LV-Kv1.3 was transfected into THP-1 cells. Following centrifugation at 1000 rpm for 5 min, the supernatant was collected and co-cultured with HK-2 cells for 48 h for subsequent assays.

MgTx, a selective Kv1.3 inhibitor, was utilized to suppress Kv1.3 expression, enabling assessment of downstream signaling alterations. To elucidate the mechanistic roles of ERK1/2 and NF-κB pathways in this context, PD98059 (a specific ERK1/2 inhibitor) and Licochalcone B (an inhibitor of NF-κB p65 phosphorylation) were applied to evaluate potential cross-talk among Kv1.3, ERK1/2 and NF-κB signaling.

### MNCs isolation from mouse peripheral blood and kidney tissues

For dissociation, the fresh mouse kidneys were cut into 1 mm^3^ pieces and placed in gentleMACSC Tube with the same amount of DMEM and digestive enzymes per the protocol of the Tissue Dissociation Kit. The tissue was dissociated using a gentleMACS Dissociator for 20 min to obtain single-cell suspensions.

The peripheral blood was treated with erythrocyte lysate, centrifuged, and resuspended in PBS to obtain a peripheral blood MNC suspension. MNCs from both blood and kidney tissues were isolated using the MNC extract kit (P6340, Solarbio, Beijing).

### Flow cytometric analysis

For flow cytometry, resuspended MNCs were blocked with FcRblock at 4 °C for 10 min and stained with anti-CD11b and anti-CD86 antibodies (cell surface staining) at 4 °C for 30 min. After washing with PBS, cells were fixed with the cell fixation buffer for 30 min at room temperature (RT). Thereafter, cells were treated with the intracellular staining permeabilization wash buffer and incubated with anti-CD206 for 30 min at RT for intracellular staining. After final washing, cells were resuspended in 500 μL of the intracellular staining buffer and analyzed using a FACS Canto II flow cytometry system (BD Biosciences). All processes are performed in the dark. The raw data were analyzed using the FlowJo_v10.8.1 software (BD Biosciences).

### Cell-based assay

Herein, 25 μL of incubated capture beads were added to all standard and sample tubes, followed by 25 μL of corresponding concentration standard or the supernatant and 25 μL of the fluorescence detection reagent. After mixing, all tubes were incubated at RT in the dark for 2.5 h. Samples were then washed with PBS and centrifuged at 1000 rpm for 5 min; the supernatant was discarded. Samples were resuspended in 500 μL of PBS and mixed thoroughly for flow cytometry. Data were analyzed using the FCAP v3.0 software, and the concentrations of cytokines in each sample were calculated based on the standard curve.

### RT-qPCR

Total RNA was extracted from the renal tissue and cultured cells using the Fast Pure Cell/Tissue Total RNA Isolation Kit per the protocol of the manufacturer. Complementary DNA was synthesized using HiScript III RT SuperMix for qPCR (+gDNA wiper). Next, the PCR reaction was performed with 20 μL sample volumes using the Taq Pro Universal SYBR qPCR Master Mix in a LightCycler 480 II thermocycler (Roche, Germany). The PCR thermocycling conditions were as follows: 95 °C for 30 s, 95 °C for 10 s, and 60 °C for 20 s (40 cycles). All primers are listed in Table 1. The 2^−ΔΔCt^ method was used to quantify the messenger RNA levels. Glyceraldehyde 3-phosphate dehydrogenase was used as the internal control.

**Table 1:** List of primers used for quantitative real-time-polymerase chain reaction analysis.

Gene name	primer (5′-3′)
mTNF-α-F	GGACTAGCCAGGAGGGAGAACAG
mTNF-α-R	GCCAGTGAGTGAAAGGGACAGAAC
miNOS-F	ATCTTGGAGCGAGTTGTGGATTGTC
miNOS-R	TAGGTGAGGGCTTGGCTGAGTG
mIL-1β-F	CTCGTGCTGTCGGACCCAT
mIL-1β-R	CAGGCTTGTGCTCTGCTTGTGA
mArg-1-F	AACCTTGGCTTGCTTCGGAACTC
mArg-1-R	GTTCTGTCTGCTTTGCTGTGATGC
mCD206-F	GTCTGAGTGTACGCAGTGGTTGG
mCD206-R	TCTGATGATGGACTTCCTGGTAGCC
mIL-10-F	CAGTGGAGCAGGTGAAGAGTGA
mIL-10-R	CCTGGAGTCCAGCAGACT CAAT
mGAPDH-F	GGCAAATTCAACGGCACAGTCAAG
mGAPDH-R	TCGCTCCTGGAAGATGGTGATGG

*qPCR* quantitative polymerase chain reaction, *TNF-α* tumor necrosis factor-α, iNOS inducible NO synthase, IL interleukin, *Arg-1* arginase-1, *GAPDH* glyceraldehyde-3-phosphate dehydrogenase.

### Western blotting

Total protein was extracted from both renal tissue and cultured cells, and protein concentrations were determined using a BCA assay kit. Equal amounts of protein (20 μg) were separated by 10% sodium dodecyl sulfate-polyacrylamide gel electrophoresis and then transferred onto 0.45- or 0.2-μm PVDF membranes. After blocking the membranes with 5% milk for 2 h at RT, they were incubated with primary antibodies (namely Kv1.3, α-SMA, TGF-β1, E-cad, p-ERK1/2, ERK1/2, p-NF-κB p65, NF-κB p65, β-Tubulin and GAPDH) overnight at 4°C. After washing, membranes were incubated with horseradish peroxidase-linked secondary antibody for 1 h at RT. Protein bands were detected and visualized on a chemiluminescence system at appropriate exposure time. Relative protein expression was quantified using the ImageJ software.

### Transwell assay

LV-Kv1.3-transfected THP-1 cells and control cells (4 × 10^4^) were seeded in the upper compartment of Transwell chambers (8 μM) containing RPMI1640 supplemented with 1% FBS. Following a 20-h incubation period, the cells were fixed with 4% paraformaldehyde for 30 min and subsequently stained with 0.1% crystal violet for 45 min, ensuring protection from light. After thorough washing, the migrated cells were observed under a microscope and analyzed using Image J software to evaluate alterations in cell migration capacity.

### Statistical analyses

Statistical analyses were performed using the Statistical Package for the Social Sciences 19.0 and GraphPad Prism 9.0 software. Data have been expressed as mean ± standard deviation. Multiple group comparisons were analyzed by one-way analysis of variance with least significance difference-t post hoc test, while pairwise comparisons utilized Student’s t-test. Statistical significance was defined as a two-tailed *P* < 0.05.

## Supplementary information

uncropped western blots

## Data Availability

The data from the current study are available upon reasonable request.
