# Supplementary material for: Therapeutic potential of voltage-dependent potassium channel subtype 1.3 blockade in alleviating macrophage-related renal inflammation and fibrogenesis
Source: Cell Death Discov. 2025 May 5;11:218. doi: 10.1038/s41420-025-02508-7 (PMC12053669; doi:10.1038/s41420-025-02508-7)

Uncropped gels for Western Blots in Figure 1E

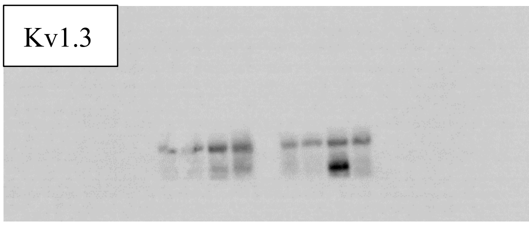

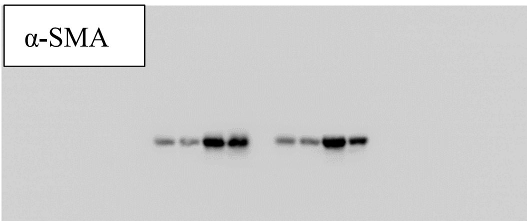

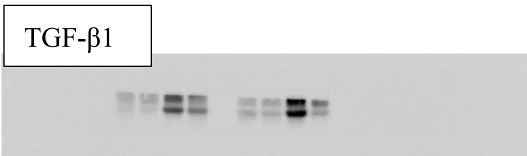

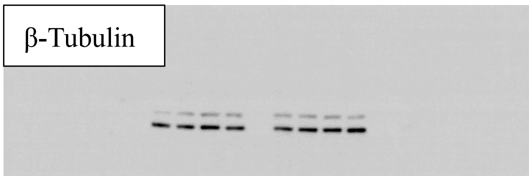

Uncropped gels for Western Blots in Figure 2C

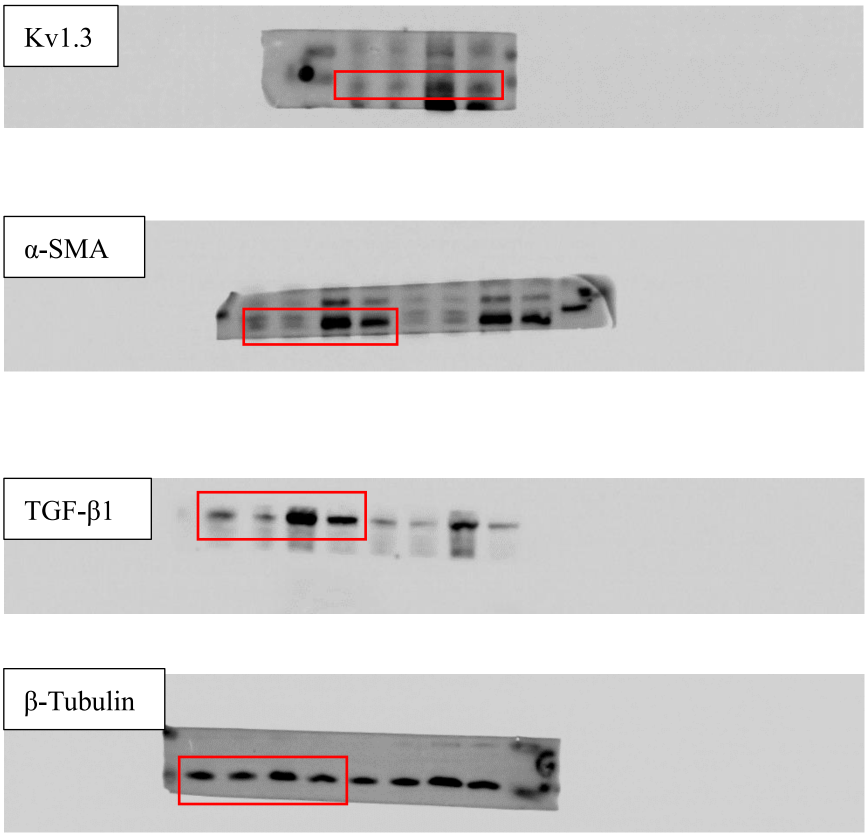

Uncropped gels for Western Blots in Figure 5B

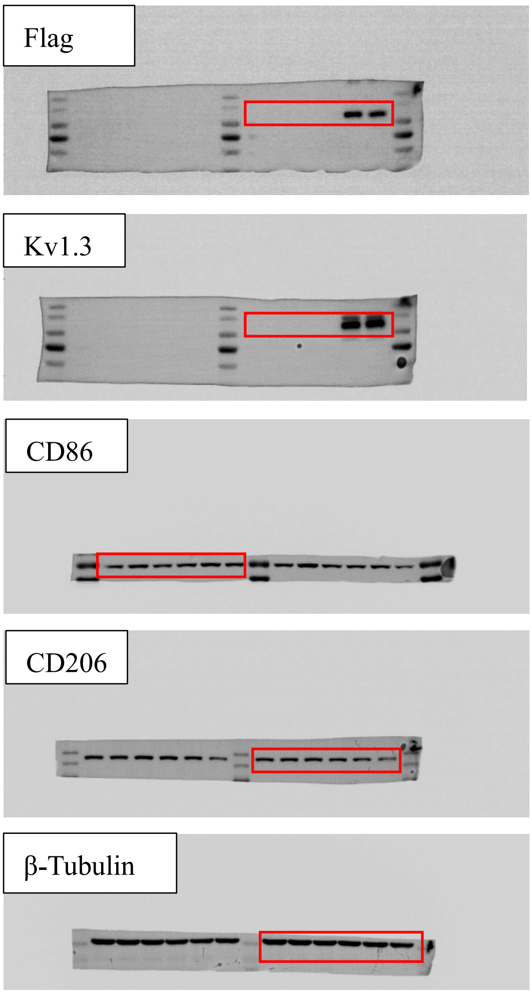

Uncropped gels for Western Blots in Figure 5F

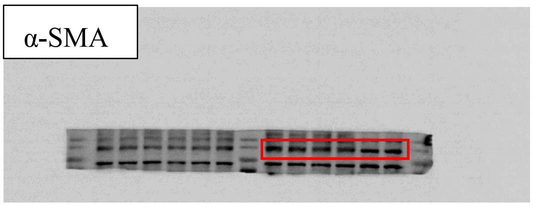

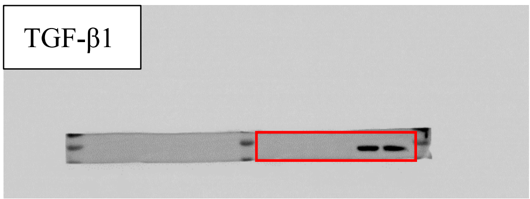

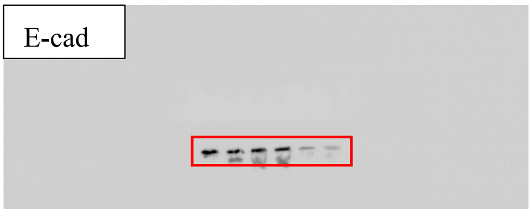

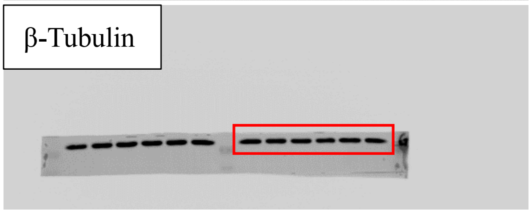

Uncropped gels for Western Blots in Figure 6A

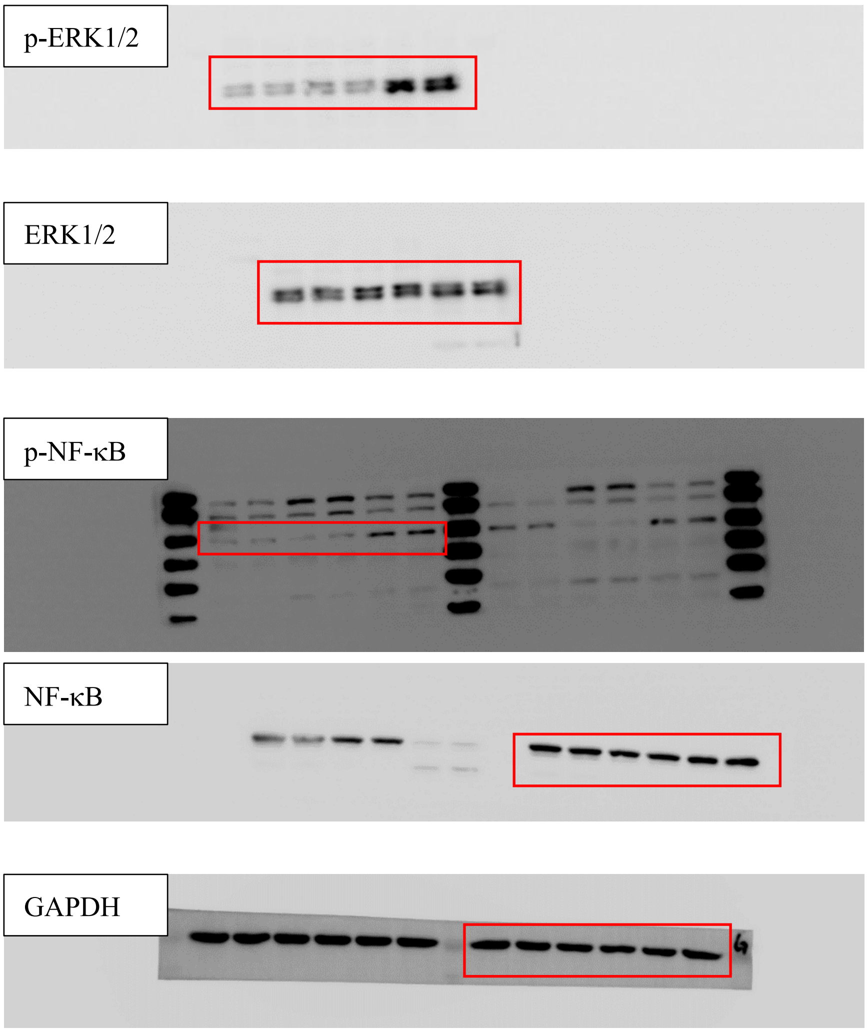

Uncropped gels for Western Blots in Figure 6C

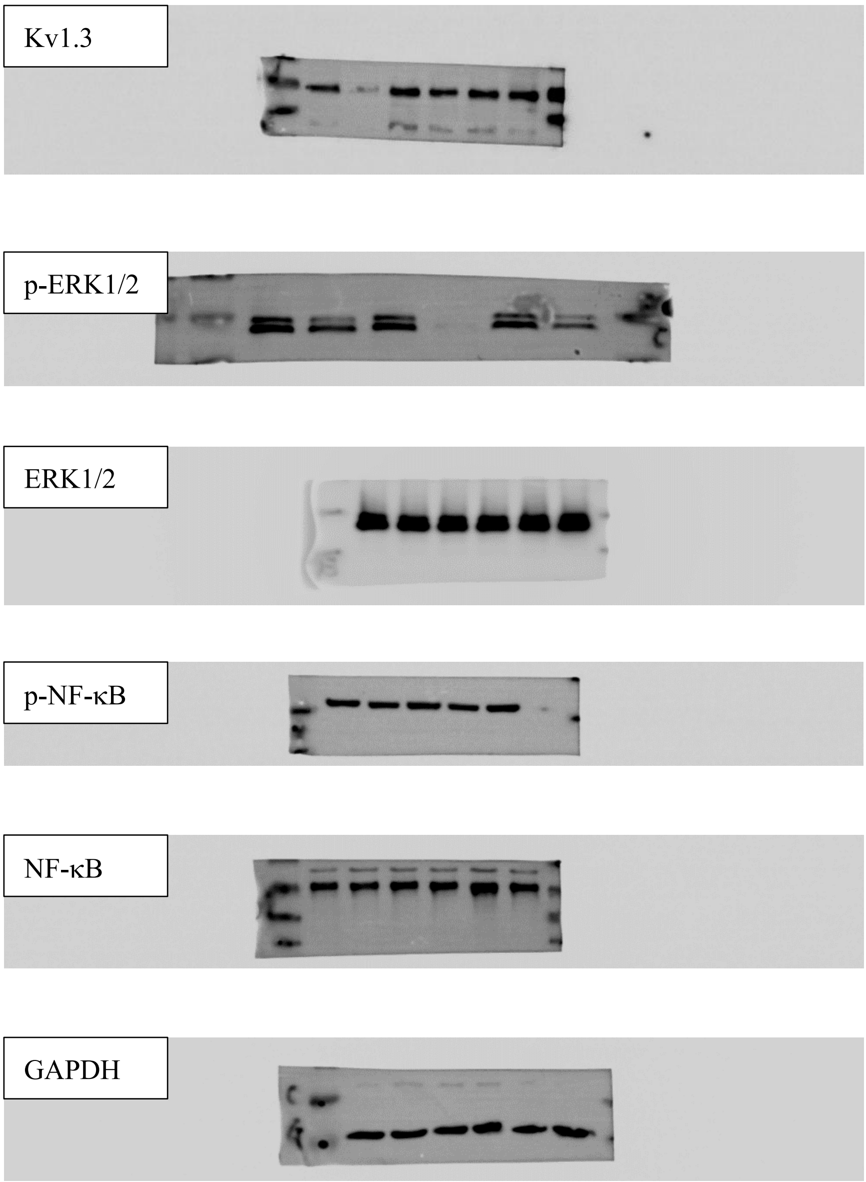

Uncropped gels for Western Blots in Figure 6D

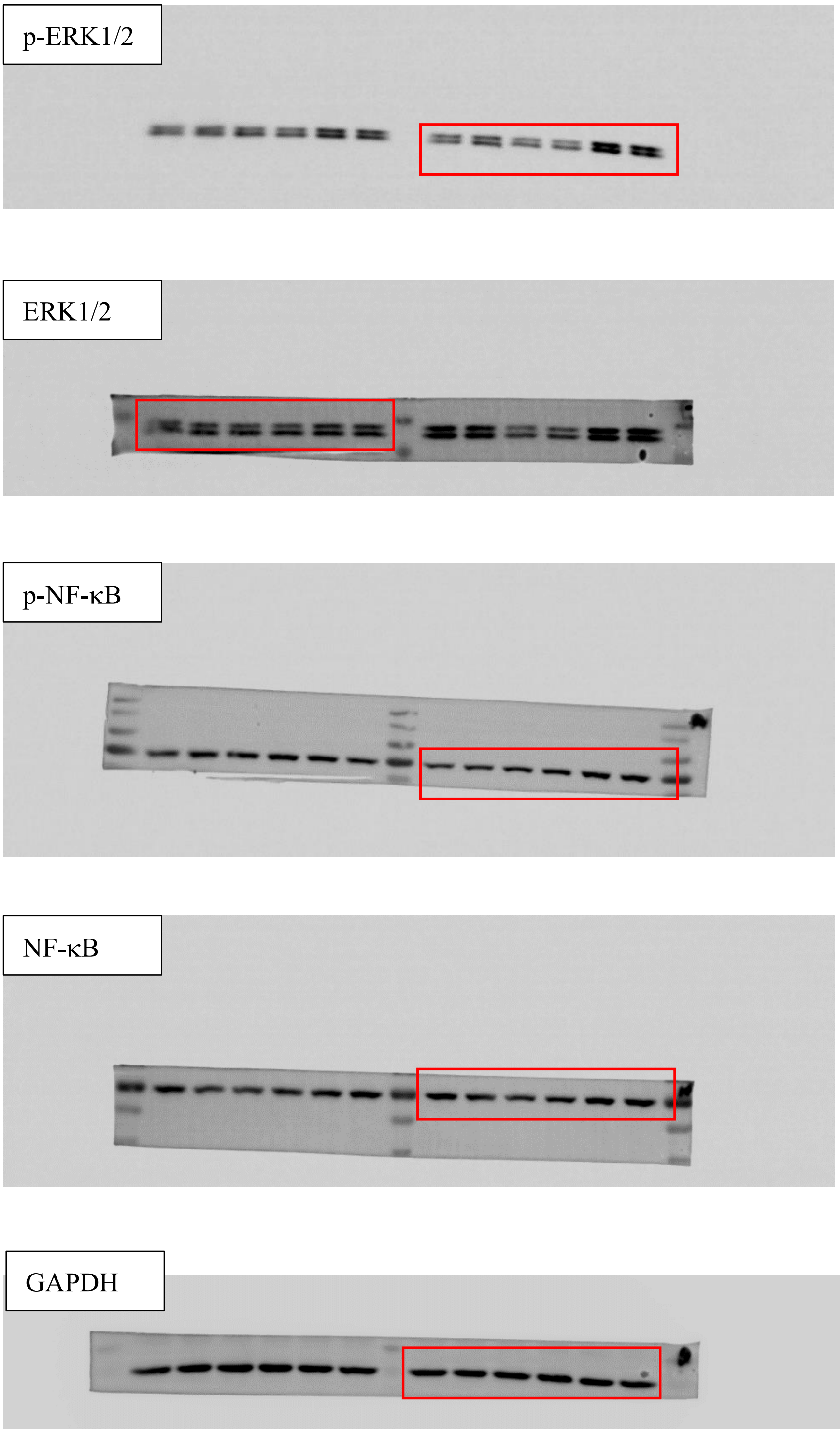

Uncropped gels for Western Blots in Figure 6E

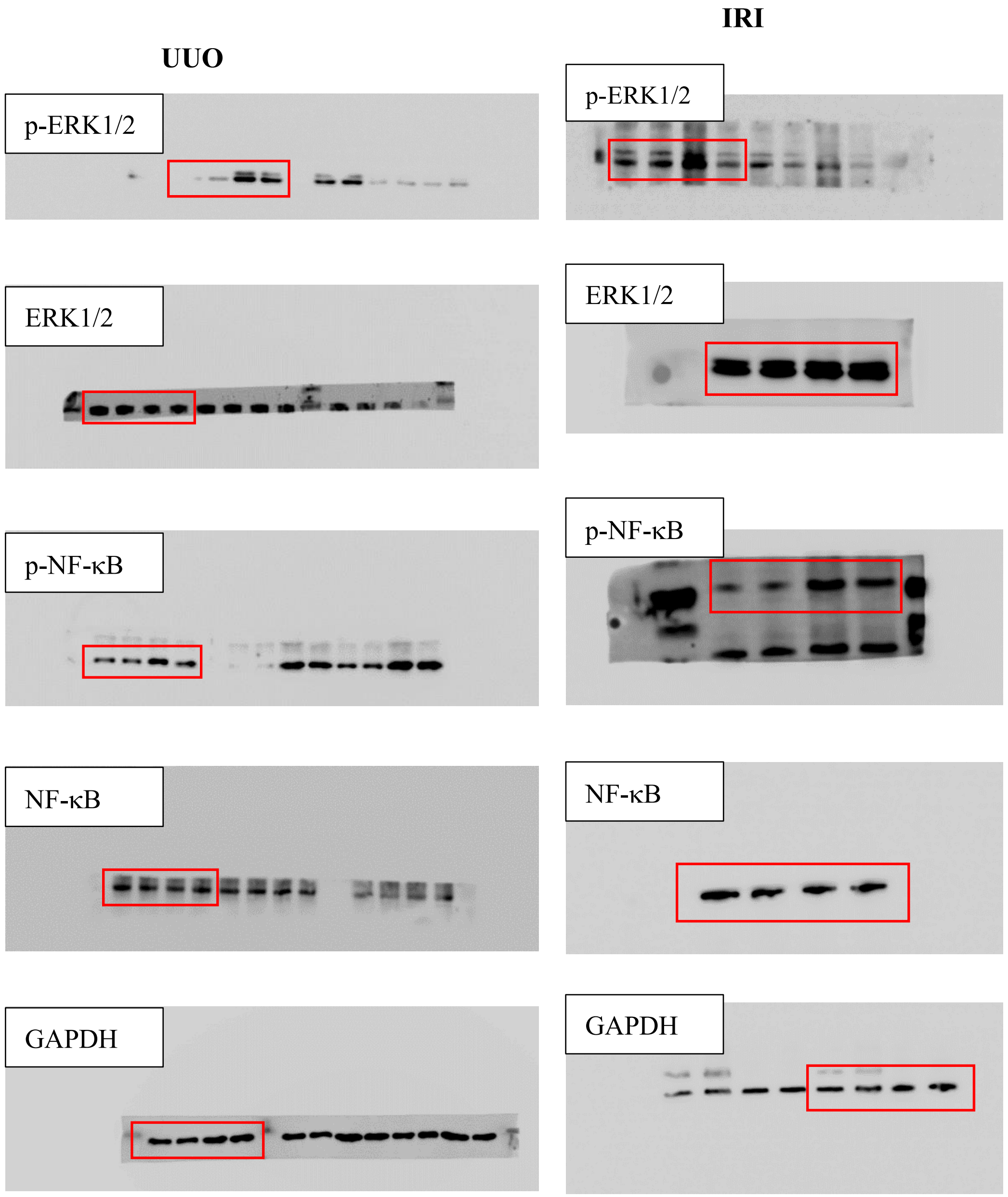

Supplement: Supplementary file 1 — uncropped western blots [file 41420_2025_2508_MOESM1_ESM.pdf]
